# Low frequency deep brain stimulation in the inferior colliculus ameliorates haloperidol-induced catalepsy and reduces anxiety in rats

**DOI:** 10.1371/journal.pone.0243438

**Published:** 2020-12-04

**Authors:** Hannah Ihme, Rainer K. W. Schwarting, Liana Melo-Thomas

**Affiliations:** 1 Behavioral Neuroscience, Experimental and Biological Psychology, Philipps-University of Marburg, Marburg, Germany; 2 Center for Mind, Brain, and Behavior (CMBB), Marburg, Germany; 3 Behavioral Neurosciences Institute (INeC), São Paulo, Brazil; Technion Israel Institute of Technology, ISRAEL

## Abstract

Deep brain stimulation (DBS) of the colliculus inferior (IC) improves haloperidol-induced catalepsy and induces paradoxal kinesia in rats. Since the IC is part of the brain aversive system, DBS of this structure has long been related to aversive behavior in rats limiting its clinical use. This study aimed to improve intracollicular DBS parameters in order to avoid anxiogenic side effects while preserving motor improvements in rats. Catalepsy was induced by systemic haloperidol (0.5mg/kg) and after 60 min the bar test was performed during which a given rat received continuous (5 min, with or without pre-stimulation) or intermittent (5 x 1 min) DBS (30Hz, 200–600μA, pulse width 100μs). Only continuous DBS with pre-stimulation reduced catalepsy time. The rats were also submitted to the elevated plus maze (EPM) test and received either continuous stimulation with or without pre-stimulation, or sham treatment. Only rats receiving continuous DBS with pre-stimulation increased the time spent and the number of entries into the open arms of the EPM suggesting an anxiolytic effect. The present intracollicular DBS parameters induced motor improvements without any evidence of aversive behavior, pointing to the IC as an alternative DBS target to induce paradoxical kinesia improving motor deficits in parkinsonian patients.

## Introduction

Patients suffering from Parkinson’s disease (PD) regularly display impairments like bradykinesia (slowness of movements) and akinesia (inability to perform voluntary movements). Drugs like L-dopa are often prescribed as first treatment [1]. However, since chronic use of L-dopa is linked to side effects like dyskinesia [2], the search for alternative treatments to gain back motor control and life quality is of major importance.

In recent years, deep brain stimulation (DBS) has provided clinical benefit for people with severe movement disorders. Especially patients with advanced PD have proved to be suitable candidates for DBS. Since PD results from a degeneration of dopaminergic cell population in the substantia nigra along with a malfunction of network activity in the basal ganglia, DBS in Parkinsonian patients typically targets motor regions of the basal ganglia [3]. However, despite such DBS can provide remarkable improvements of cardinal Parkinsonian symptoms, an increasing number of patients report side effects from DBS surgery as well [4]. For instance, DBS of the subthalamic nucleus, the preferred target, although significantly improving bradykinesia, has also been demonstrated to exacerbate dyskinesia [5], and to impair verbal fluency [6] and cognition in some patients [7]. DBS of the ventral nucleus of the thalamus or the globus pallidus can even induce mood disorders or suicidal ideation [8]. In this respect, studies using animal models and experimental DBS can be valuable in order to clarify DBS mechanisms aiming to minimize side effects and search for new DBS targets.

We have shown that high-frequency DBS in the inferior colliculus (IC), a midbrain structure classically related to auditory processing, can serve as an alternative DBS target to ameliorate motor deficits in akinetic rats [9,10]. The IC is an important relay station for ascending and descending auditory information but is distinguished from other brainstem auditory nuclei by its indirect projections to motor pathways [11]. The IC has long been implicated as part of the brain aversive system since its electrical stimulation induces defensive behaviors such as arousal, freezing and escape responses (an explosive motor behavior), that mimic reactions to threatening environmental stimuli [12,13]. Using the haloperidol-induced catalepsy model in rats [14], which resembles akinesia in Parkinsonian patients, we have demonstrated that this kind of explosive motor behavior induced by intracollicular DBS can also be elicited in cataleptic rats, thereby releasing the cataleptic state [9]. Such behavioral outcome is reminiscent of a clinical phenomenon known as paradoxical kinesia, which is defined as the sudden and transient ability to perform fluent voluntary movements in response to an emotionally significant trigger (acoustic/visual stimuli) [15–17].

However, although the attenuating effect of intracollicular DBS on catalepsy is desirable, the aversive and anxiogenic side effect induced by this stimulation limits its use in clinical settings. Aiming to overcome this limitation, in the present study we searched for the effective combination of DBS parameters that induces motor improvement while avoiding negative emotional side effects. It is known from other DBS targets that the stimulation effect varies depending on the choice of stimulation parameters, such as current amplitude, frequency and pulse width [18,19]. Thus, in the present study, 30 Hz frequency stimulation was chosen because clinical evidence suggests that lower DBS frequencies might be more effective than higher ones [20]. This is particularly the case for structures located outside the basal ganglia, such as the pedunculopontine nucleus, another brainstem structure involved in motor behavior, where optimal stimulation frequencies range between 20–180 Hz [21]. In addition, we choose current amplitudes from 200 to 600 μA based on a previous pilot study showing that no aversive behavior was induced when these amplitudes were combined with 30 Hz frequency applied in the IC. Finally, we investigated these parameters in two different time schedules such as intermittent or continuous stimulation, based on existing DBS protocols used during electrode implantation in patients during which a fixed frequency is combined with a voltage increasing [22].

Thus, in the present study, we hypothesized that by changing DBS parameters in the IC, the anxiogenic effect would be avoided while the motor improvement would be preserved. For that, we established an efficacious combination of DBS parameters that induces paradoxical kinesia while avoiding negative emotional side effects.

## Materials and methods

### Animals

Male Wistar rats (n = 31, Charles-River, Germany) weighing 300-350g were grouped in cages of five each. After surgery, rats were kept individually for one day and were later housed in pairs in Macrolon type III cages with extra high acrylic covers (L: 22 cm x W: 38 cm). Laboratory conditions were standardized (23° C temperature, 40–60% humidity, 12/12 day/night cycle) and free access to water and food was provided. All protocols were following the current European guidelines and approved by the ethics committee of the local government (Regierungspräsidium Gießen, TVA G53-2016). Four rats were excluded from the catalepsy study due to technical reasons and three rats dropped out of the EPM.

### Electrode description and implantation

Rats were implanted with a microelectrode unit, consisting of a stimulation electrode (90% platinum, 10% iridium wire; core diameter 125μm, outer diameter 150 μm, impedance <10 kOhm; Thomas RECORDING GmbH, Giessen, Germany), connected to a contact plate and a platinum wire reference electrode (shaft diameter, 100 μm). Rats were anaesthetized with 2% isoflurane (Baxter Deutschland GmbH Germany) and fixed in a stereotactic frame (TSE Systems, Bad Homburg, Germany). Ophthalmic ointment Bepanthen (Bayer Vital GnbH, Leverkusen, Germany) was applied to prevent eye drying. The rat was placed in a stereotactic apparatus and after receiving 0.5 ml of xylazine (s.c.; Dentsply De Trey GmbH, Konstanz), a midline scalp incision was made and small craniotomies were performed. Then, stainless steel screws were fixed into the skull and the electrode was inserted into the IC using the following coordinates from the brain atlas by Paxinos and Watson [23] with lambda as reference: antero-posterior = + 1.0 mm, medio-lateral = + 1.5 mm, dorso-ventral = + 4.5 mm. The electrode and the screws were covered with ultraviolet adhesive (smart-fix, LUX-Tool) and synthetic resin. A protective cap for the implantable electrode unit was used to cover the electrode contacts. Finally, the rats received buprenorphine (Titolare A.I.C., Berkshire, UK) at a dose of 0.05mg/kg (i.p.) to minimize discomfort and they were kept under surveillance until waking up.

### General test protocol

**Fig 1:**
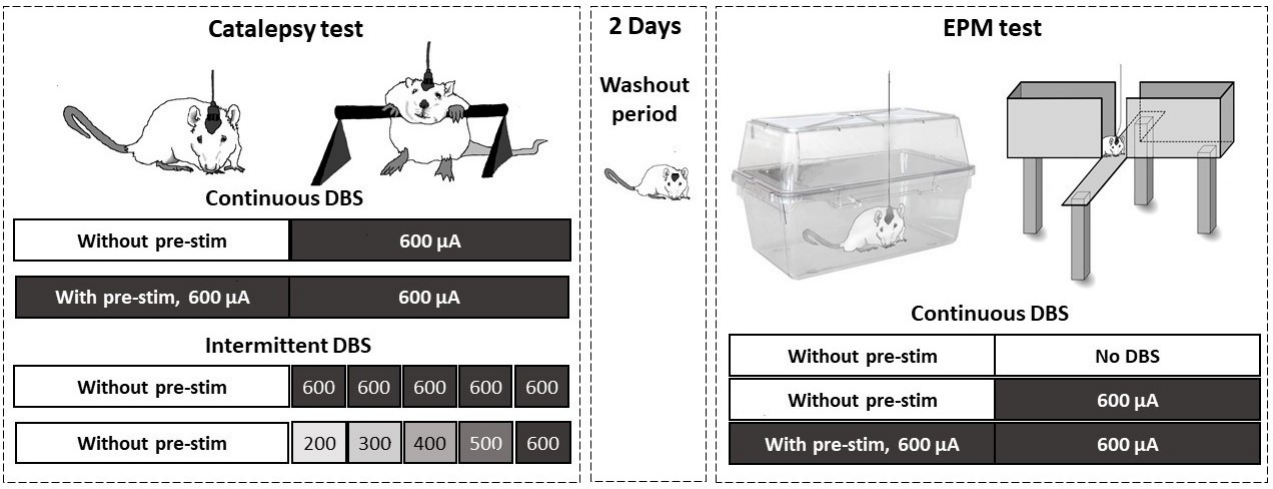
An overview of the test protocol. Behavioral testing started after a one-week recovery period and consisted of catalepsy test and EPM test. All rats were randomly assigned to the following groups: continuous DBS with or without pre-stimulation and intermitted DBS with 200–600 μA or 600 μA current amplitude and submitted to the catalepsy test. After a washout period all rats were reassigned to no DBS (sham group), continuous DBS with and without pre-stimulation groups and submitted to the elevated plus maze (EPM) test. Each rat was tested three times, two times for catalepsy (intermittent and continuous DBS) and once in the EPM. Rat assignment to the groups was randomized throughout the whole study.

**Fig 2:**
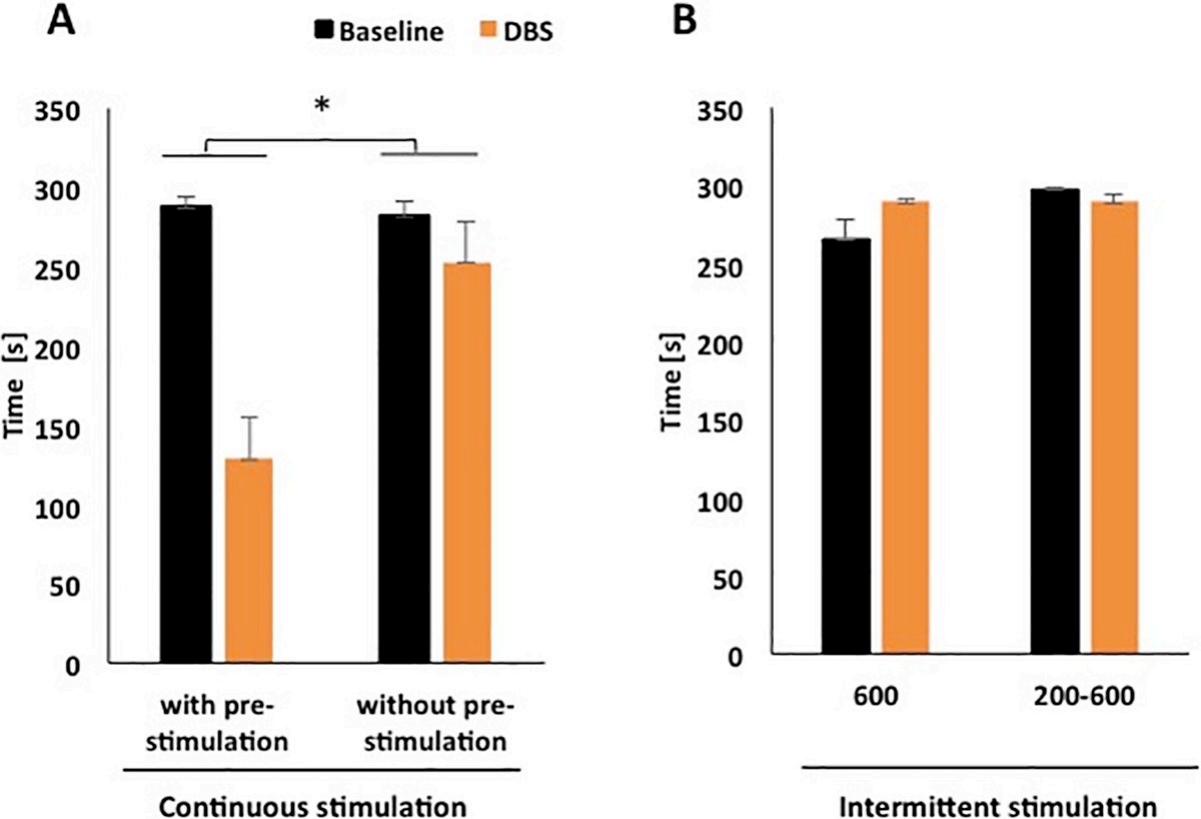
**(A)** Shows the mean catalepsy times during baseline and DBS for continuous and (**B**) intermittent groups. Rats from intermittent DBS groups received either 5 x 60 s (30 s interval) 30 Hz and 600 μA current amplitude or 5 x 60 s (30 s interval) 30 Hz and 200–600 μA current amplitude. Rats assigned to continuous DBS groups received either no pre-stimulation or pre-stimulation during 5 min and then, stimulation during the catalepsy test for additional 5 min. For these groups, DBS consisted in 30 Hz frequency, 600 μA current amplitude. * p<0.05.

**Fig 3:**
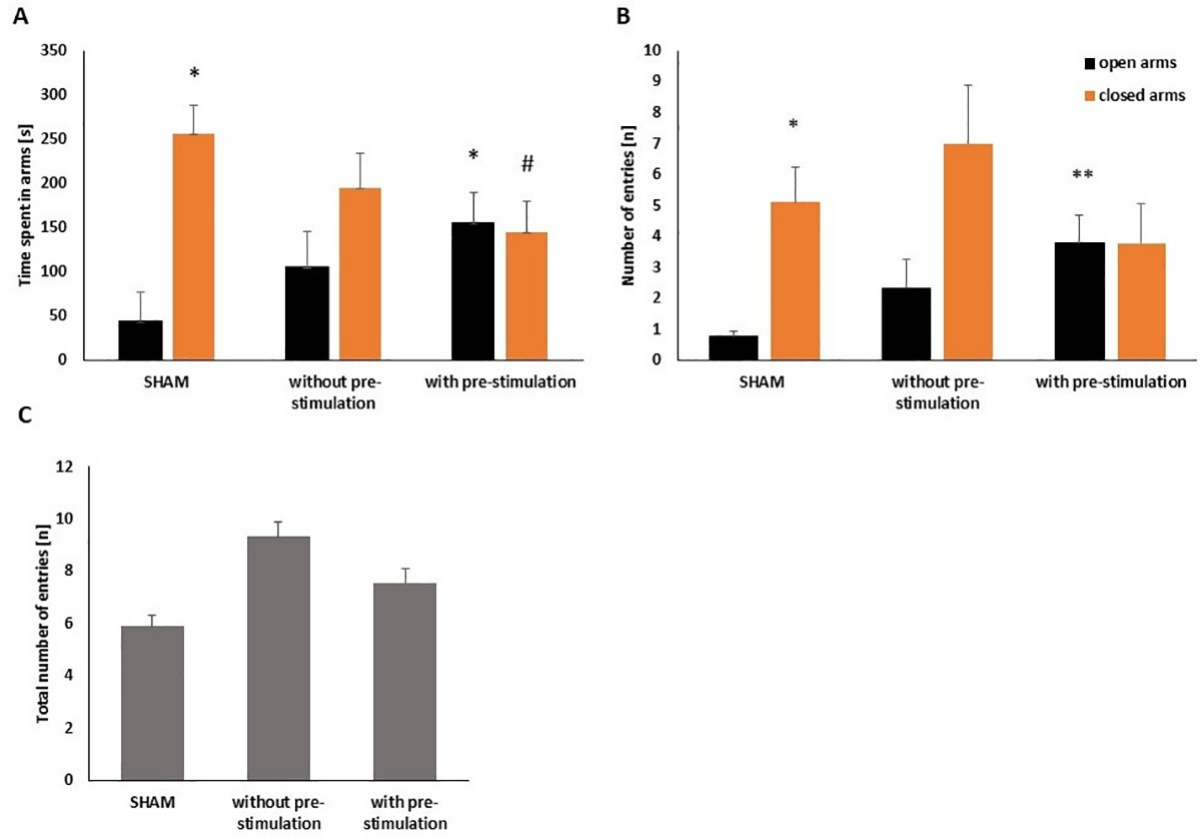
Effect of intracollicular DBS on exploratory behavior in the EPM test. **(A)** Shows time spent, **(B)** numbers of entries into the open and closed arms of the EPM and **(C)** total number of entries into the arms of each experimental group. All rats were constantly connected with their implanted electrode to the current generator via cable. Rats were assigned to the following groups: SHAM group that did not receive any stimulation; continuous DBS without pre-stimulation group that received DBS only during the EPM test and continuous DBS with pre-stimulation group that received continuous stimulation both in the home cage (5 min) and during the EPM test. Rats belonging to SHAM group spent less time and entered less into the open arms showing a classical pattern of anxiogenic-like behavior. Both groups receiving continuous intracollicular DBS showed no preference for the open or closed arms, showing a clear anxiolytic effect of DBS. Groups did not differ significantly from each other in the total amount of entries in arms (p = 0.446) indicating that the present DBS treatment did not induce any kind of unusual motor activity that could affect the results. * p<0.05 and ** p<0.01 related to SHAM open arms; ♯ p<0.01 related to SHAM closed arms.

Behavioral testing started after a one-week recovery period and consisted of the catalepsy test and the EPM test (see Fig 1). First, for the catalepsy test, the rats were randomly assigned to the following groups: **intermittent DBS with 200–600μA** (n = 15) receiving current amplitude from 200 up to 600 μA at 100 μA steps (60 s each with 30 s intervals between stimulation periods); **intermittent DBS with 600μA** (n = 12) receiving the same number of trials (5 x 60 s with 30 s intervals) but with a constant amplitude of 600 μA. The rats were randomly reassigned to the following groups: **continuous DBS with pre-stimulation** (n = 13) receiving continuous DBS with an amplitude of 600μA for 5min before and 5 min during catalepsy test; and **continuous DBS without pre-stimulation** (n = 9) receiving DBS 600μA for 5 min only immediately after being placed on the bar (see Fig 2). A stimulation frequency of 30 Hz with a pulse width of 100 μs was used for all DBS procedures. Stimulation duration of 5 min was chosen based on our previous studies in rats [10] and also on studies in humans showing an overall motoric improvement after 5 min DBS in the subthalamic nucleus [24]. To assess the effect of intracollicular DBS on anxiety, these same rats were tested on the EPM. For that they were randomly reassigned to three experimental groups: In the **SHAM** control group (n = 9), rats did not receive any electrical stimulation. Rats assigned to the **continuous DBS with pre-stimulation** group (n = 10) were continuously stimulated in their home cage 5 min immediately before and then also during the EPM test. Rats from **continuous DBS without pre-stimulation** group (n = 9) were submitted to the same procedure except that the stimulation was switched off when they were in the home cage (see Fig 3). The rats was tested two times for catalepsy (once in intermittent and once in continuous stimulation condition) in a counterbalanced way and once for EPM test, with a washout period of 48 h.

### Catalepsy test

#### Drug and dose

Catalepsy was induced by haloperidol (0.5 mg/kg; Janssen Pharmaceutica, Beerse, Belgium) diluted in physiological saline and administered i.p. in a volume of 1.0 ml/kg.

#### Test setup

Testing was performed in an arena (40 x 40 x 40 cm), under red light (~30 lux) and was recorded by a video camera positioned centrally 1m above the arena. In order to assess catalepsy, a horizontal bar (height 8 cm) was placed centrally into the arena [14].

#### Procedure

The bar test started with the baseline assessment. For that purpose, rats were placed with their forepaws on the bar approximately 40 min after receiving haloperidol injection. There, the step-down latency was recorded with a cut-off time of 5 min. The baseline catalepsy time was taken only when a given rat remained at least 120 s on the bar. If a given rat did not meet this criterion, testing was repeated 10 min later. The step-down-latency of the first successful trial was considered as baseline indicating the strength of the drug-induced catalepsy. DBS procedure started immediately after taken the baseline. For this, the stimulation electrodes were connected to a pulse generator using a tethered system (STG3008-FA, Multichannel Systems, Germany) and the rats were placed again on the arena. Forthwith, the stimulation procedure started except for the SHAM group that did not receive any stimulation and the step-down-latency was assessed.

### Elevated Plus Maze (EPM) test

#### Test setup

The EPM comprised two open arms (50 x 10 cm) and two enclosed arms (50 x 10 cm, with 40 cm high walls) with a central square connecting the four arms elevated 50 cm above the floor. Testing was performed under red light (~30 lux) and recorded by a video camera fixed above the central square of the EPM.

#### Procedure

A cable from the pulse generator was connected to the previously implanted stimulation electrode and the rat was placed back into a home cage that was positioned next to the generator and the EPM, for 5 min. Then, the rat was placed onto the EPM for 5 min while the electrode stayed connected to the generator via the cable. The cable was fixed to a motile stick ensuring complete mobility and normal exploratory behavior. Rats belonging to with pre-stimulation group received DBS when in both home cage and EPM, while rats from without pre-stimulation group received DBS only during EPM test. In case of the sham stimulation group, the pulse generator was kept off. Only continuous DBS was used, since this stimulation was efficient to reduce catalepsy time.

#### Behavioral assessment

For the EPM test, the number of entries into the open and closed arms and the time spent in the open and the closed arms were assessed. “Entering an arm”was defined by the animal being with all four paws in the respective arm [25].

#### Statistical analysis

Statistical analysis was conducted with IBM SPSS 20, while the graphics were processed with Sigma Plot. A repeated measures ANOVA was performed to analyze the catalepsy time. Kruskal-Wallis-Test was conducted to determine if there was a significant difference among time spent and entries number in the arms in EPM. Pairwise comparisons were conducted by using the Wilcoxon-Test. The alpha levels of the pairwise comparisons were adjusted according to the Bonferroni-Holm method.

#### Perfusion and histology

After behavioral testing, all rats received an overdose of pentobarbital (Fagron GmbH & Co, Germany; 600 mg/kg). When breathing stopped, the same stimulation cable used during the behavioral experiments was connected to the implanted electrode and electrical stimulation (current intensity 50 μA, pulse width: 100 μs; pulse interval: 100 μs) was applied for 90 s in order to produce a small lesion around the electrode tip. This step was crucial to better localize electrode tip placement during the later histological analysis. Then, all rats were perfused intracardially with physiological saline solution and 4% paraformaldehyde. The brains were removed and stored in fixation solution at 4°C. The brain slices were cut with a cryostat (Model 1850, Leica) and stained with cresyl violet. The position of the electrodes was identified according to the brain atlas of Paxinos and Watson [23].

## Results

### Intracollicular DBS reduced haloperidol-induced catalepsy

A repeated measures ANOVA showed a significant difference in the mean catalepsy times for continuous stimulation [F(1, 20) = 7.703, p = 0.012; Fig 2A] but not for intermittent stimulation groups [F(1, 25) = 3.204, p = 0.086; Fig 2B].

### Effect of intracollicular DBS in the EPM test

The Kruskal-Wallis-test revealed that intracollicular DBS significantly affected the time spent in the open arms [H(2) = 7.784, p = 0.020]. Pairwise comparisons showed that the group continuous DBS with pre-stimulation spent significantly more time in the open arms compared to the SHAM group (p = 0.016; Fig 3A). There was no difference regarding the time spent in the open arms when comparing continuous DBS without pre-stimulation and SHAM (p = 0.380) group or continuous DBS with and without pre-stimulation (p = 0.625) groups. Concerning the time spent in the closed arms, a significant difference was found between groups [H(2) = 7.784, p = 0.020]. Rats which received continuous DBS with pre-stimulation spent significantly less time (p = 0.008) in the closed arms than those of the SHAM group. There was no significant difference between continuous DBS without pre-stimulation and SHAM (p = 0.380) groups nor between continuous DBS with and without pre-stimulation (p = 0.625) when comparing the times spent in the closed arms. Noteworthy, only rats from the SHAM group spent significantly less time in the open arms than in the closed arms (p = 0.040), showing an anxiogenic-like behavior expected for control condition. In contrast, there was no significant difference when comparing the times spent in the open and closed arms for the groups continuous DBS without pre-stimulation (p = 0.139) or continuous DBS with pre-stimulation (p = 0.767; see Fig 3A), which indicates a clear anxiolytic-like effect of DBS, since rats showed no preference for any of the arms.

Also, significant group differences in the number of entries into the open arms [H(2) = 9.118, p = 0.010] were observed. Pairwise comparisons revealed that rats from continuous DBS with pre-stimulation group entered more often into the open arms than rats of the SHAM group (p = 0.008). In contrast, there was neither a significant difference between SHAM and continuous DBS without pre-stimulation (p = 0.227) nor between continuous DBS with and without pre-stimulation (p = 0.661). Importantly, comparisons within groups showed that only rats of the SHAM group entered less into the open arms as compared to the closed arms (p = 0.013). There was no difference between the number of entries into the open and closed arms for the continuous stimulation without pre-stimulation (p = 0.123) or continuous stimulation with pre-stimulation groups (p = 0.917; Fig 3B). Last, DBS did not induce any kind of unusual motor activity since the total number of entries (sum of entries into the open and closed arms; Fig 3C) did not differ significantly [H(2) = 1.615, p = 0.446] among the groups.

### Histological analysis

**Fig 4:**
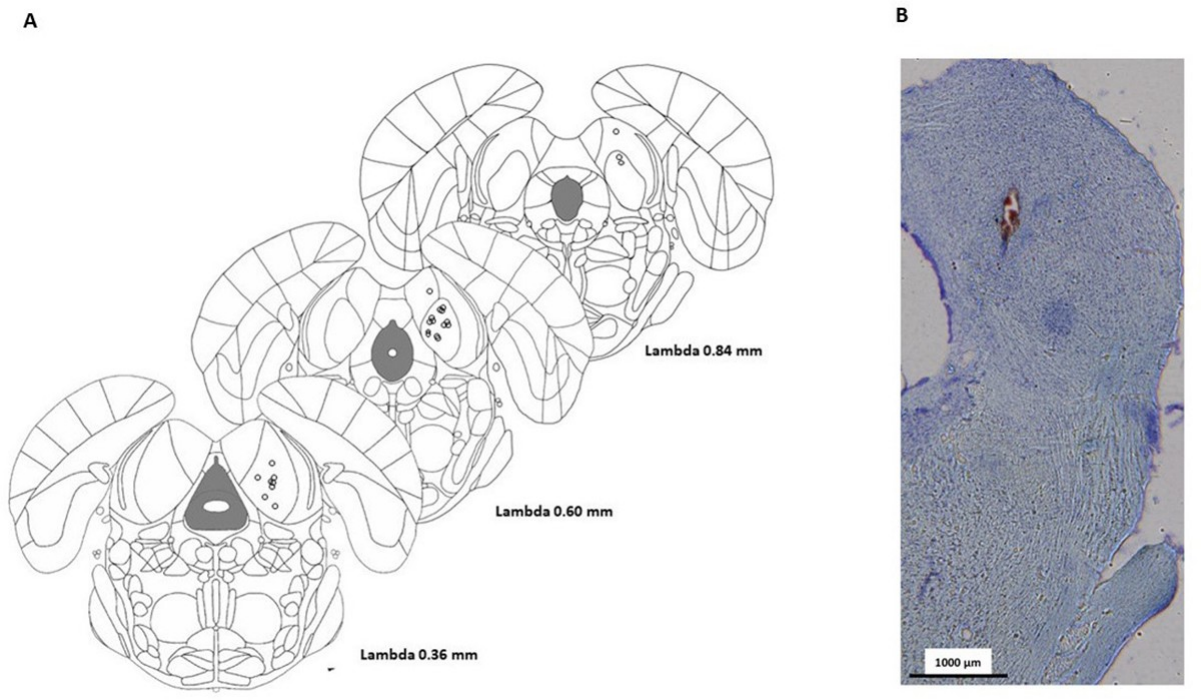
**(A)** White circles represent electrode placements in coronal sections from the Paxinos and Watson’s atlas. All electrode tips were located within the IC. Not all sites of stimulation are visible because several dots overlap; **(B)** exemplary brain section exhibiting the tip of the electrode within the IC.

The final post-mortem histological analysis showed that all the electrodes tips were placed within the central nucleus of the IC (Fig 4).

## Discussion

In the present study, we demonstrated that intracollicular DBS (30Hz) can improve catalepsy induced by systemic haloperidol. We found that only rats that received continuous stimulation with pre-stimulation showed a significant reduction in catalepsy time compared to the ones receiving continuous DBS without pre-stimulation or intermittent stimulation. Additionally, these stimulation parameters delivered in the IC did not induce any kind of aversive behavior in rats exhibiting catalepsy induced by haloperidol. The fact that using these intracollicular DBS parameters the motor improvement effect was preserved while an aversive effect was not evident points to this structure as a potential alternative DBS target to treat akinesia, corroborating our general proposition. Here it is important to highlight that intermittent stimulation in the present study was based on existing DBS protocols used during electrode implantation in PD patients. During this procedure a fixed frequency is combined with a voltage increasing in order to find the ideal stimulation parameter for each patient, although after the surgery DBS is applied continuously during everyday activity [22]. The present data showing that intermittent DBS led to a different behavioral outcome as compared to continuous DBS (with the same stimulation parameters) suggest that, at least in case of the IC this protocol used for the electrode implantation is not appropriate.

In order to investigate systematically any kind of aversive side effect, rats were submitted to the EPM test under continuous DBS stimulation. Only the stimulation parameters effective in reducing catalepsy time were subsequently used during EPM test. Here, it is important to highlight that rats received no drug treatment and similar to the previous DBS tests of this study, DBS delivered in the IC did not induce any kind of aversive behavior (such as jumping or running). Beyond that, continuous DBS with or without pre-stimulation induced an anxiolytic-like effect in the EPM test. While the sham group spent more time in the closed arms than in the open arms exhibiting a clear anxiogenic-like behavior, rats receiving continuous DBS with or without pre-stimulation showed no preference for any arm, notably a clear anxiolytic-like effect. Importantly, the total number of entries did not differ significantly among groups suggesting that the present DBS parameters did not induce any kind of unusual motor activity that could have affected the results. The significant increase of time spent and the number of entries into the open arms induced by DBS in the present study is comparable to the effect of clinically effective anxiolytics such as chlordiazepoxide or diazepam as shown by Pellow et al [25]. Furthermore, rats receiving continuous DBS with pre-stimulation during the EPM test displayed a significantly higher number of head dipping and rearing compared to all other groups (see supporting information). These results are not only interpreted as an indication of the integrity of exploratory behavior but also as an index of less anxiety [26], supporting the assumption of an anxiolytic-like effect in rats receiving continuous DBS in the IC. This is especially relevant, as anxiety disorders often co-occur with PD [27].

To the best of our knowledge, this is the first study showing that DBS in the IC induces a clear anxiolytic effect. More than that, this is probably also the first study showing that only by changing DBS parameters in the IC, the behavioral outcome switches from anxiogenic to anxiolytic. DBS mechanisms underlying the anxiolytic effect observed in the present study remain unclear. The fact that opposite effects can be obtained stimulating the same site at the same structure only by using different stimulation parameters, raises the question whether this is also the case for other brain structures. Indeed, opposing effects of DBS have already been demonstrated when the electrode is placed in different sites at the same structure [27–29], or when it is misplaced [30], which is not our case.

Once the same DBS parameters that induce anxiolytic effect also reduces catalepsy when applied in the IC, help us to understand the mechanisms underlying paradoxical kinesia induced by relevant emotional stimulation. Indeed, the motor improvement observed in the present study is interpreted as paradoxical kinesia, an interesting clinical phenomenon during which otherwise akinetic PD patients can regain the ability to perform fluent voluntary movements, as for instance quickly catching a ball or running, when exposed to emotional or external (acoustic/visual) stimuli [16,17]. In the present study akinesia was induced by a high dose of the typical neuroleptic haloperidol, which blocks striatal dopaminergic D2 receptors, leading to a state referred to as catalepsy. Since akinesia/bradykinesia as observed in parkinsonian patients could be dependent on their emotional state [15] we previously investigated whether presenting emotionally (aversive or appetitive) relevant stimuli could induce paradoxical kinesia in rats. In these studies we have demonstrated that aversive [9] or apetitive intracollicular DBS [10] or even appetitive auditory stimulation [31] temporarily interrupting haloperidol-induced catalepsy, representing an animal model of paradoxical kinesia. Although we still do not know which is the exact role of the emotional aspect of the trigger in producing paradoxical kinesia, our present and previous studies [9,10,31] point to the IC as a key structure since it processes emotional/sensory information and influences motor output [32].

First attempts to adjust the current parameters in order to induce paradoxical kinesia while avoiding aversive side effects were made recently by our group [10]. This study was performed in two steps: (1) pre-test session, the current amplitude was individually defined at the aversive threshold (the lowest current intensity that produced aversive behavior such as running, or jumping) combined with a very high-frequency (2500 Hz); (2) test session, the individual current amplitude previously defined at the aversive threshold was combined with a lower frequency (30 Hz) during the behavioral tests. In that study we showed that this last combination of DBS parameters did not cause any aversive/explosive behavior while preserving the motor improvement [10], but we did not test the rats for possible anxiogenic effects. The data presented here represent a completely new study and a clear step forward since (i) the induction of aversive behavior in order to define stimulation parameters was avoided, which is clinically relevant; (ii) we obtained an anxiolytic effect of DBS, which might be clinically relevant as well; (iii) the motor improvement effect was preserved; (iv) a refinement of DBS parameters was obtained since the results show that continuous stimulation with at least 5 min pre-stimulation is better than intermittent stimulation. Together, our previous [9,10,31] and the present data corroborates our hypothesis that the IC is involved in the elaboration of paradoxical kinesia regardless of its important role in the brain aversive system [12,13].

Beyond that one can speculate that paradoxical kinesia induced by intracollicular DBS in the present study is dependent on dopamine levels. Indeed, it has been shown that electrical stimulation in the IC can lead to an increase of extracellular dopamine in the frontal cortex [33]. However, further research on this mechanism is needed since some authors argue that dopamine release is not responsible for the occurrence of paradoxical kinesia [34]. Reinforcing the participation of the IC in paradoxical kinesia, we have previously shown that GABAergic and glutamatergic intracollicular neural substrates modulate haloperidol-induced catalepsy in rats [35–37]. Despite that, the IC-DBS mechanisms may differ completely from those. Yet, the present results point to the IC as an important sensorimotor interface attenuating both anxiety and haloperidol-induced catalepsy.

In summary, we found effective intracollicular DBS parameters in order to improve catalepsy induced by haloperidol and these effect where obtained without aversive side effect; rather, an anxiolytic effect was obtained in rats. Finally, the present study strengthens our proposition that the IC can be a potential alternative DBS target to improve motor deficits.

## Supporting information

S1 File (DOCX)
