# Supplementary material for: Low frequency deep brain stimulation in the inferior colliculus ameliorates haloperidol-induced catalepsy and reduces anxiety in rats
Source: PLoS One. 2020 Dec 4;15(12):e0243438. doi: 10.1371/journal.pone.0243438 (PMC7717509; doi:10.1371/journal.pone.0243438)
Supplement: S1 File — (DOCX) [file pone.0243438.s001.docx]

**Supporting information**

Title S1: **Intracollicular deep brain stimulation of parkinsonian rats ameliorates motor deficits and anxiety**

*Hannah Ihme, MSc^1^, Rainer K. W. Schwarting, PhD^1,2,^, Liana Melo-Thomas, PhD^1,2,3*^*

*^1^Behavioral Neuroscience, Experimental and Biological Psychology, Philipps-University of Marburg, Gutenbergstrasse 18, 35032 Marburg, Germany*

*^2^Center for Mind, Brain, and Behavior (CMBB), Hans-Meerwein-Straße 6, 35032 Marburg, Germany*

*^3^Behavioral Neurosciences Institute (INeC), Av. do Café, 2450, Monte Alegre, Ribeirão Preto, 14050-220, São Paulo, Brazil.*

**^*^**Corresponding author

**Corresponding author’s address:**

Liana Melo-Thomas, PhD

Experimental and Biological Psychology, Behavioral Neuroscience, Faculty of Psychology, Philipps-University of Marburg, Gutenbergstraße 18, 35032 Marburg, Germany.

Phone: +55 (0)6421 2823694

email: [melothom@staff.uni-marburg.de](mailto:melothom@staff.uni-marburg.de)

**S2: Supplementary methods and results**

**Exploratory Behaviour**: During the the EPM test, the numbers of the following behavioral categories were measured: a) head-dipping: protruding the head beyond the edge of an arm and scrutinizing vertically downward; b) scanning: protruding the head over the edge of an arm and scrutinizing horizontally; c) rearing: rising on the hind limbs; d) grooming: cleaning any part of the body surface with the tongue, teeth, and/or forepaws; and e) end-exploring: number of times the rat reached the end of an proximal or distal arm [S1].

**Statistical analysis:** The groups differed significantly in the amount of head dipping [H(2)=10.532), p=0.005] and rearing [H(2)=12.854, p=0.002] shown in the EPM (Table 1). More precisely, pairwise comparisons showed that only the rats belonging to the group continuous DBS with pre-stimulation performed significantly more head dipping (p=0.004) and rearing (p=0.002) compared to the SHAM group. Accordingly, there was no significant difference regarding the number of head dipping (p>0.05) or rearing (p>0.05) neiter when comparing SHAM and continuous DBS without pre-stimulation nor when comparing continuous DBS with and without pre-stimulation. The Kruskal-Wallis-Test indicated no significant differences between groups for stretched attendance [H(2)=0.205, p=0.903], risk assessment [H(2)=2.744, p=0.254], end exploring [H(2)=5.014, p=0.082], scanning [H(2)=4.849, p=0.089] or grooming [H(2)=2.000, p=0.368].

|  | SHAM | Continuous DBS without pre-stimulation | Continuous DBS with pre-stimulation |
| --- | --- | --- | --- |
| Head dipping | 5.89 **±** 1.51 | 10.44 **±** 2.28 | 18.33 **±** 2.54* |
| Rearing | 0.11 **±** .11 | 1.00 **±** .88*§* | 3.67 **±** 1.62*♯* |
| Stretched attendance | 3.44 **±** .71 | 3.78 **±** .85 | 4.56 **±** 1.23 |
| Risk assessment | 8.78 **±** 1.61 | 6.56 **±** 1.75 | 4.56 **±** 1.48 |
| End exploring | 0.78 **±** .46 | 2.11 **±** 1.07 | 4.22 **±** 1.26 |
| Scanning | 3.33 **±** 1.52 | 7.00 **±** 2.75 | 11.44 **±** 3.12 |
| Grooming | 0.00 **±** 0.00 | 0.00 **±** 0.00 | 0.11 ± 0.11 |

**S1 Table 1.** *Effect of intracollicular DBS on exploratory behaviors during the EPM test. Groups differed significantly in the amount of head dipping and rearing behaviors. Pairwise comparison showed a significant difference in head dipping between SHAM and continuous DBS with pre-stimulation (* p=0.005), a significant difference in rearing between SHAM and continuous DBS with pre-stimulation (♯ p<0.005) and continuous DBS without and with pre-stimulation (****§*** *p=0.016).*

S3 Reference:

S1. Cruz, A. D. M., Frei, F. and Graeff, F. G. Ethopharmacological analysis of rat behavior on the elevated plus-maze. *Pharmacol. Biochem. Behav.* 1994;***49*(1)**: 171-176.
